# Delayed recovery and host specialization may spell disaster for coral‐fish mutualism

**DOI:** 10.1002/ece3.10209

**Published:** 2023-06-22

**Authors:** Catheline Y. M. Froehlich, O. Selma Klanten, Martin L. Hing, Mark Dowton, Marian Y. L. Wong

**Affiliations:** ^1^ Faculty of Science, Medicine and Health University of Wollongong Wollongong New South Wales Australia; ^2^ School of Life Sciences University of Technology Sydney Sydney New South Wales Australia

**Keywords:** coral‐dwelling gobies, habitat specificity, host plasticity, multiple disturbances, mutual symbioses, mutualism

## Abstract

Mutualisms are prevalent in many ecosystems, yet little is known about how symbioses are affected by ecological pressures. Here, we show delayed recovery for 13 coral‐dwelling goby fishes (genus *Gobiodon*) compared with their host *Acropora* corals following four consecutive cyclones and heatwaves. While corals became twice as abundant in 3 years postdisturbances, gobies were only half as abundant relative to predisturbances and half of the goby species disappeared. Although gobies primarily occupied one coral species in greater abundance predisturbances, surviving goby species shifted hosts to newly abundant coral species when their previously occupied hosts became rare postdisturbances. As host specialization is key for goby fitness, shifting hosts may have negative fitness consequences for gobies and corals alike and affect their survival in response to environmental changes. Our study is an early sign that mutualistic partners may not recover similarly from multiple disturbances, and that goby host plasticity, while potentially detrimental, may be the only possibility for early recovery.

## Graphical abstract

Multiple disturbances are breaking down the mutualisms between corals and coral‐dwelling fishes. Predisturbances, coral gobies occupied a variety of coral host species, and most corals were occupied. Postdisturbances, gobies were slower to recover than their coral hosts, although the few goby species that remained had switched to newly available coral host species. Three years after disturbances were not enough time for goby recovery as most corals remained unoccupied and were small.

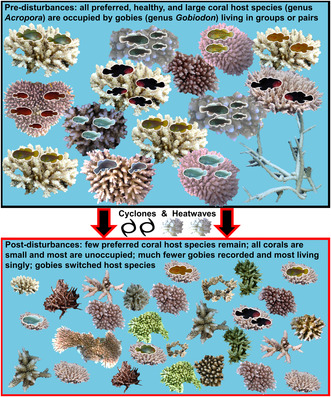

## 1 INTRODUCTION

In the face of climate change, multiple consecutive disturbances are becoming increasingly prevalent globally, and ecosystem stability is being threatened as a result (Hughes et al., 2018; Turner, 2010). Relationships between organisms are important for maintaining ecosystem balance and diversity during these challenging times, especially when one of these organisms is a habitat‐forming foundation species, for example, conifers, kelps, and corals (Angelini et al., 2011; Denton & Gokhale, 2019). Mutually beneficial symbioses (here termed “mutualisms”) often promote the survival of foundation and partner species, but anthropogenic disturbances are adding extreme pressures on these relationships (De Fouw et al., 2016; Denton & Gokhale, 2019). A key question to arise is: will organisms in mutualisms respond similarly to consecutive disturbances, and what factors are important in the persistence of both partners (Marquis et al., 2014)?

For symbioses in which one organism relies on the other for limiting resources like food and shelter, the host species is a key determinant of the fitness of its symbiotic partner (mediated through growth, feeding, and reproductive advantages) (Hughes et al., 2000; Munday, 2001). The benefits that the host incurs from their symbiotic partner may also vary with the species of the partner, for example, specialized nutrients and protection (Douglas, 1998; Kiers et al., 2010; Sensenig et al., 2017). However, as disturbances are intensifying and occurring more frequently, some host species are being disproportionally affected than other hosts (Bonin, 2012; Douglas, 1998; Kiers et al., 2010). In response, symbiotic partners may leave their host if it becomes unhealthy (Pratchett et al., 2020; Sensenig et al., 2017), or they may stay and facilitate their mutual recovery (Chase et al., 2018; Kiers et al., 2010; Marquis et al., 2014).

On coral reefs, corals are host to many mutually symbiotic organisms, such as microbes, *Symbiodinium* algae, crabs, and coral‐dwelling fishes (McKeon et al., 2012; Munday et al., 1999; Thompson et al., 2015). These inhabiting symbiotic partners often specialize on particular host coral species, which they may leave or stay during environmental stress (Bonin, 2012; McKeon et al., 2012; Munday et al., 1999; Thompson et al., 2015). Little is known about how climate change affects these mutualisms and the degree of host specialization by inhabiting taxa, despite the importance of these ecological partnerships. For example, coral‐fish symbioses are important for coral health because inhabiting fish improve coral growth and protect corals from toxic algae, sedimentation, predation, and stagnant hot water build‐up (Chase et al., 2018; Chase, Pratchett, McWilliam, et al., 2020; Dirnwoeber & Herler, 2013; Dixson & Hay, 2012; Holbrook et al., 2008; Lassig, 1981). Often, coral‐dwelling fishes specialize on different hosts and vary to what extent they are specialized, which commonly can be categorized with those fish species that live only in 1–3 coral species (host specialist) versus those that use 4–11 coral species (host generalist) (Bonin, 2012; Caley & Munday, 2003; Munday, 2001), here defined as coral richness specificity. Host specialization can also be defined as the extent to which one fish species will use one coral species over another, which can be calculated along a continuum (i.e., specificity continuum) or categorized based on a specific threshold (i.e., proportional coral specificity), for example, using a single coral species at least 75% of the time (host specialist) versus <75% of the time (host generalist). Host specialization by coral‐dwelling fishes likely affects how both symbiotic partners recover given that climatic disturbances affect some hosts more than others (Froehlich et al., 2021; Hughes et al., 2019). Since fish provide important services for the health and growth of corals, the ability of fish to shift hosts may result in some coral hosts becoming unoccupied and thus more vulnerable to disturbances.

Coral‐dwelling gobies from the genus *Gobiodon* provide an excellent model for assessing how host use may affect their mutualisms because these gobies are site‐attached. Once individuals settle as larvae, gobies remain within the coral branches and do not leave their coral host, except for some extreme circumstances regardless of life stage like their coral host dies, they get evicted by conspecifics, or their mate dies, and they need to find new mates (Bonin et al., 2009; Munday et al., 1998; Nakashima et al., 1996; Wong & Buston, 2013). If gobies are displaced, for example by storm activity or predator attempt, they will return to their coral host instead of using alternative corals, even with high predation risk (Froehlich et al., 2022). The choice of host is important for gobies, as well as for other coral‐dwelling fish, and strong competition among goby species exists for specific hosts (Munday, 2001; Pereira et al., 2015). Compared with other coral‐dwelling fishes that move among or above coral branches (Chase, Pratchett, & Hoogenboom, 2020; Rueger et al., 2014, 2018), most goby species reside deep within the branches of corals and often rest directly on the branches using their fused pelvic fin for suction (V. Y. M. Froehlich pers. obs.). The movement, feeding efficiency, and growth of gobies are thus related to the coral species they inhabit because different species of corals vary in several physical characteristics like size, shape, and interbranch space and chemical characteristics like stinging defense (Ben‐Ari et al., 2018; Caley & Munday, 2003; Gardiner & Jones, 2005; Munday, 2001; Pereira & Munday, 2016; Untersteggaber et al., 2014). Some goby species also grow bigger on certain coral species than others, highlighting the fitness benefits of certain coral species (Munday, 2001; Pereira & Munday, 2016). The type of coral host that gobies inhabit therefore plays an important role in the ecology of gobies.

Here, our 7‐year study (2013–2020) shows that coral‐dwelling gobies (genus *Gobiodon*) either disappeared or shifted their occupation of host corals (genus *Acropora*) after an unprecedented succession of disturbances with limited recovery periods: two category four cyclones (2014, 2015) and two prolonged heatwaves (2016, 2017), which caused extensive coral bleaching. We compared their host specialization using three metrics: specificity continuum (continuous), proportional coral specificity (categorical), and coral richness specificity (categorical). Previous studies have shown trade‐offs between goby fitness and host specialization, with particular coral hosts improving the growth and survival of specialist gobies compared with generalist gobies (Caley & Munday, 2003; Munday, 2001). Accordingly, the shifts in host occupation (i.e., host plasticity) coupled with a lag in recovery of gobies will likely hamper the fitness of both parties during the crucial and early stages following disturbances (Chase et al., 2018; Chase, Pratchett, & Hoogenboom, 2020; Chase, Pratchett, McWilliam, et al., 2020; Dirnwoeber & Herler, 2013; Dixson & Hay, 2012; Penin et al., 2010). By surveying gobies and their coral hosts before and after each disturbance, and then 3 years postdisturbances, we found that gobies fared far worse than corals, with a distinct time lag in the early signs of recovery of gobies compared with corals.

## 2 METHODS

### 2.1 Study location

**FIGURE 1:**
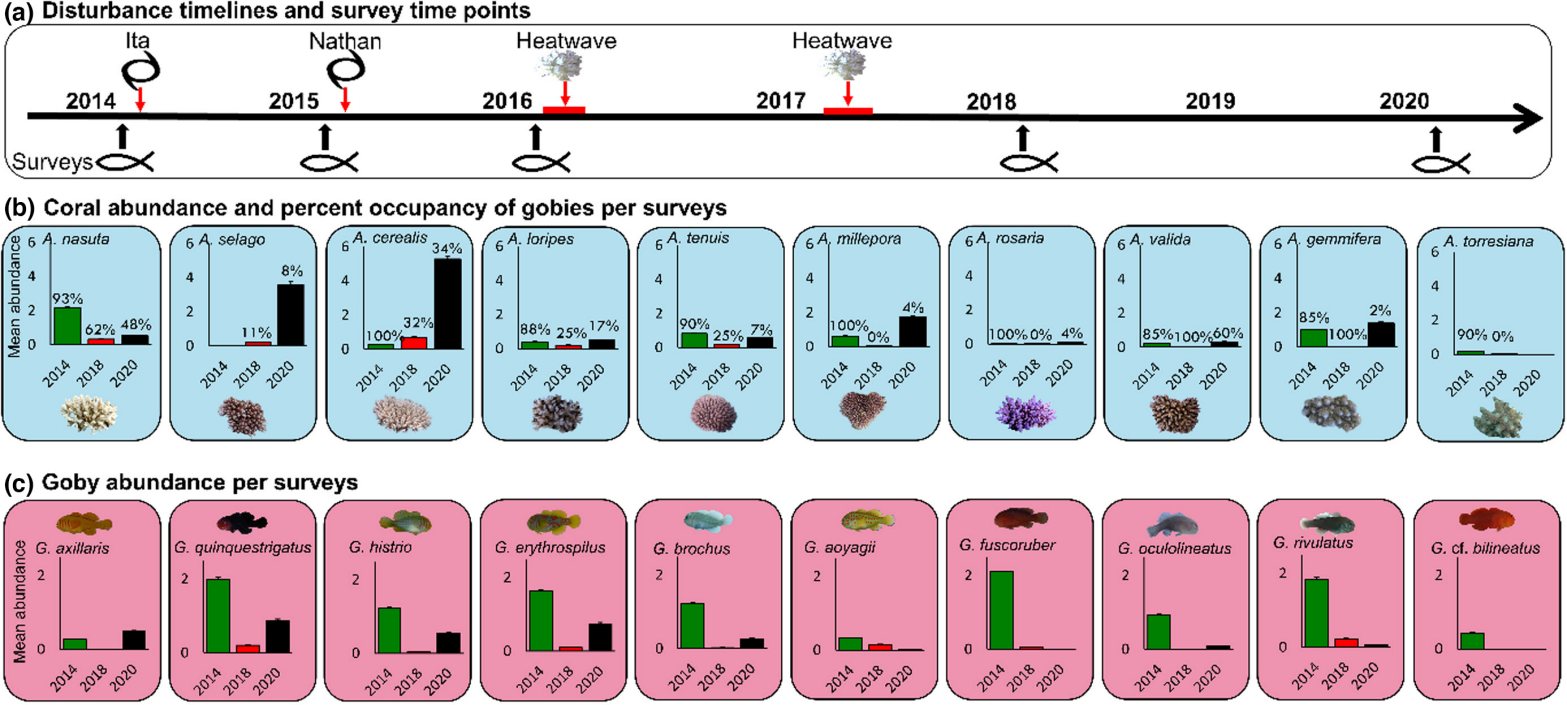
Multiple disturbances changed the mean abundance per transect of *Acropora* corals (blue) and their symbiotic *Gobiodon* gobies (red). (a) Following consecutive disturbances (two cyclones and two heatwaves), (b) the 10 most common coral hosts, and (c) their goby symbionts experienced drastic changes in abundance. There were no changes in abundances after the first two disturbances (i.e., each cyclone); however, there were significant changes after the last disturbances (i.e., both bleaching events), and thus, we display changes postdisturbances. Error bars are standard error. Percentages above bars represent the proportion of corals that were occupied by gobies during that particular survey year.

All sampling was completed at reef sites within Lizard Island, Great Barrier Reef, Queensland, Australia (−14.687264, 145.447039, Appendix F1). Lizard Island was affected by four extreme climatic events annually from 2014 to 2017: cyclone Ita (category 4) in April 2014, cyclone Nathan (category 4) in March 2015, a heatwave causing a mass‐bleaching event from March to April 2016, and a second heatwave causing a mass‐bleaching event from February to May 2017. Sites were visited before these events in February 2014 (*n* = 18 sites), after the first cyclone in January–February 2015 (*n* = 16), after the second cyclone in January–February 2016 (*n* = 19), after both heatwaves in February–March 2018 (*n* = 22), and 3 years after the last disturbance in January–March 2020 (*n* = 24) (Figure 1a). Not all sites were sampled each year due to weather conditions and scouring effects of cyclones that left some sites with only bare rock.

### 2.2 Sampling method

Surveys were completed at each time point for the presence of *Gobiodon* goby spp. (using Munday et al., 1999 for nominal species identification) within *Acropora* coral spp. (using Veron et al., 2018 for nominal species identification). There were two types of surveys used: (1) in 2014 (*n* = 61 transects at 18 sites), 2018 (*n* = 40 transects at 16 sites), and 2020 (*n* = 50 at 18 sites), corals were surveyed 1 m on either side of 30‐m transects and were repeated at the same GPS points each sampling event, although not all transects were repeated during each sampling event; and (2) in 2015 (*n* = 108 at 16 sites) and 2016 (*n* = 140 transects at 19 sites), corals were surveyed 1 m on either side of 4‐m cross‐transects at different GPS points but within the same sites (Froehlich et al., 2021; Hing et al., 2018). Transects were repeated at the same GPS points within sites, although not all transects were repeated during each sampling event: In addition, since very few corals were encountered along transects after the four disturbances, random searches occurred in 2018 (*n* = 28 at 18 sites) and 2020 (*n* = 34 at 19 sites). When a live *Acropora* coral was encountered, the coral was measured and averaged along its width, length, and height (Kuwamura et al., 1994). Only corals at least 7 cm in average diameter were included in surveys because smaller corals were never found occupied by gobies (Froehlich et al., 2021). The coral was searched for a *Gobiodon* species using a bright torch light (Bigblue AL1200NP), and the species and number of individuals were noted. Individuals were identified as adults or juveniles based on coloration and size. The study was completed under the animal ethics protocols AE1404 and AE1725 from the University of Wollongong, and research permits G13/36197.1, G15/37533.1, and G18/41020.1 issued by the Great Barrier Reef Marine Park Authority.

### 2.3 Data analysis

For changes in coral and goby populations, we used data from transects only since random searches did not follow any particular transect techniques. The following variables had many zero data points per transect after multiple disturbances, and accordingly were compared among survey yr (fixed factor) and site (random factor) with a generalized linear mixed model (GLMER: Poisson family) using a zero‐inflated model: coral richness and abundance, adult goby richness and abundance, and juvenile goby richness and abundance. Note: for all abundance variables, only line transects in 2014, 2018, and 2020 were used to remove transect‐type bias in abundances. The following variables were compared among survey yr (fixed factor) and site (random factor) with linear mixed models (LMER): average coral diameter, coral occupancy (whether occupied or unoccupied by *Gobiodon* spp.), and adult goby group size (juveniles were not included because they were observed moving between coral heads). All analyses were completed in R (v3.5.2) (R Core Team, 2018) with the following packages: tidyverse (Wickham et al., 2019), lme4 (Bates et al., 2015), lmerTest (Kuznetsova et al., 2017), LMERConvenienceFunctions (Tremblay & Ransijn, 2020), piecewiseSEM (Lefcheck, 2016), glmmTMB (Brooks et al., 2017), emmeans (Lenth et al., 2020), DHARMa (Hartig & Lohse, 2020), performance (Lüdecke et al., 2021), and plotly (Sievert, 2020). Coral and goby communities for the 10 most common species of each genus were compared among survey yr (fixed factor) and site (random factor) with a permutational analysis of variance (PERMANOVA) in Primer‐E software (v7).

For host specialization analyses, we used data from transects and random searches. Data for particular species were removed for years in which the species was observed <8 times in order to allow for enough observations to assess host specialization use. Three out of the 13 goby species observed in the surveys were excluded from host specialization analysis since they were consistently too rare (*G. citrinus*, *G. okinawae*, and *G*. sp. *D*). The corals inhabited per goby species were then combined per year. Coral species inhabited were compared among goby species (fixed factor) and survey yr (fixed factor) using PERMANOVA. The three different host specialization metrics were included in the analysis depending on whether the metric was continuous or categorical. For the continuous metric of specificity continuum (proportion of occurrences in which only one coral species was used per goby species [continuous variable, 0–1]), the metric was added to the analysis as a covariable, which was calculated from the first survey predisturbances (2014). The other two host specialization metrics were included as factors and we also added a factor addressing the sociality of gobies as some goby species primarily live in groups whereas other primarily live in pairs: PERMANOVAs were repeated (without the covariable as it is correlated with the following factors) to individually include each of the following explanatory factors calculated from the first survey predisturbances (2014): coral richness specificity (fixed factor, host specialization category per goby species on the basis that goby conspecifics used up to three coral species [specialist] versus more than 3 coral species [generalist]), proportional coral specificity (fixed factor, host specialization category per goby species on the basis that 75% or more goby conspecifics used a single coral species [specialist] versus <75% of gobies used a single coral species [generalist]), and whether goby species were social (living in groups) or asocial (living in pairs) as identified in Hing et al., 2018 (fixed factor: asocial or social). Note: the goby species factor was nested within each of the factors in the later PERMANOVAs.

## 3 RESULTS

### 3.1 Goby recovery is lagging behind the recovery of their coral hosts

Throughout these consecutive disturbances and 3 years postdisturbances, we surveyed 36 species of *Acropora* coral hosts used by 13 species of coral‐dwelling gobies (*Gobiodon*) known to occur at Lizard Island, Great Barrier Reef, Australia (−14.687264, 145.447039, Figure 1a). Less than 1 year after the last disturbance (2018), coral and goby abundances, richness, coral diameter and occupancy, and goby group size were at an all‐time low (Appendix F2, *p* < .001, see Appendix T1 for all statistical results). Three years postdisturbances (2020), there were signs of recovery for corals as coral abundance and richness were higher than previously recorded, but corals remained extremely small and were rarely occupied by gobies (Appendix F2). Goby richness and abundances were still very low, and gobies continued to occur singly, whereas they were living in pairs or in groups predisturbances (Appendix F2). However, the number of juvenile goby species and their abundance improved (Appendix F2).

We focused specifically on the abundance of the 10 most commonly used coral hosts and 10 most common goby species and found that not all goby and coral species responded in the same way. Abundances were different among years (*p* < .001, Figure 1b), with eight coral species becoming extremely rare after disturbances, which was not surprising because 50% of the transects lacked corals compared with only 5% before disturbances (Froehlich et al., 2021). However, there was recovery 3 years postdisturbances when only 17% of transects lacked corals. Surprisingly, two coral species became more abundant immediately after disturbances even though they were rare before (*A. cerealis* and *A. selago*). These species became at least 10 times more abundant 3 years postdisturbances than predisturbances (Figure 1b). In general, more corals were found without goby partners post‐ compared with predisturbances (Figure 1b).

For gobies though, it was a different story. Only one goby (*G. axillaris*) returned to its predisturbance abundance in 2020 (i.e., had fully recovered), whereas all other species either became too rare/no longer sighted or were only at 50% predisturbance abundances (Figure 1c). Three species disappeared altogether from our survey sites immediately postdisturbances in 2018 (*G*. cf. *bilineatus*, *G. fuscoruber*, and *G. oculolineatus*), and an additional two species (*G. aoyagii* and *G. rivulatus*) disappeared 3 years postdisturbances in 2020 (Figure 1c). Of those species that disappeared, three were already rare before disturbances, but one was originally the most common species surveyed (*G. fuscoruber*).

### 3.2 Some gobies showed plasticity in their host specialization

**FIGURE 2:**
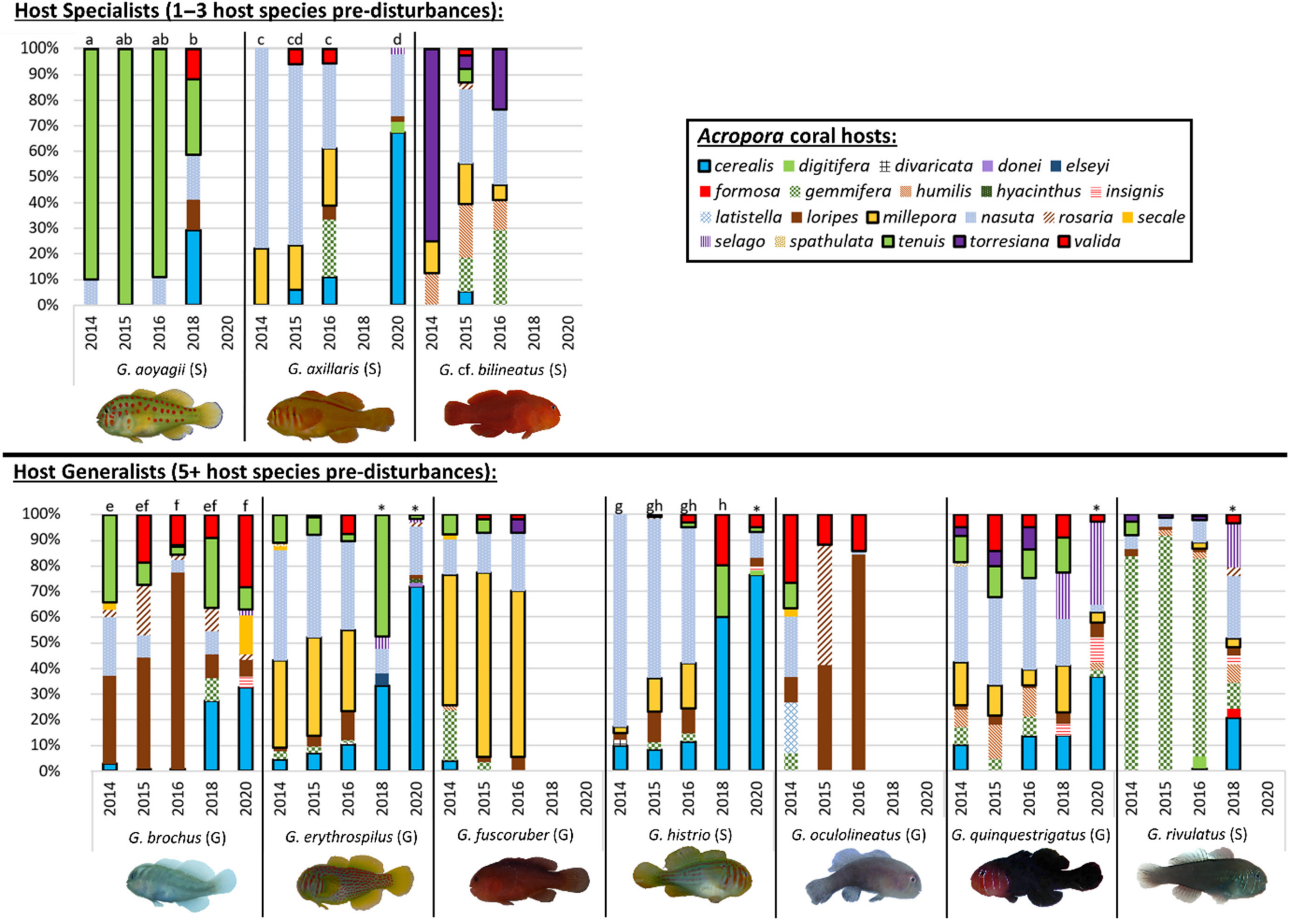
Host specialization of *Gobiodon* gobies in *Acropora* coral hosts changed following multiple disturbances. Proportion of all *Acropora* species used by the 10 most common *Gobiodon* species from surveys: predisturbances (2014), after cyclone Ita (2015), after cyclone Nathan (2016), after two back‐to‐back heatwaves/bleaching events (2018), and 3 years postdisturbances (2020). Goby species are separated into two categories using the coral richness specificity metric of host specialization as calculated predisturbances. Acronyms next to goby species represent two categories using the proportional coral specificity metric of host specialization: “S” describes host specialists using the same coral species >75% of the time predisturbances, and “G” describes host generalists using the same coral species <75% of the time predisturbances. Letters above each bar represent host use differences among sampling years that are significantly similar to one another within species, and asterisks represent host occupation that is significantly different from all others within a species. If there are no bars, the species was no longer observed or too rare (*n* < 8). If there are no letters above the bars, these bars were not significantly different from one another for that goby species.

Host specialization was compared using three different metrics. Predisturbances, each goby species usually inhabited a range of coral species with minimal overlap among goby species (*p* < .01), but gobies changed their range of coral hosts throughout the climatic disturbances (*p* < .01, Appendix F3, Figure 2). Not all gobies responded the same in terms of host occupation throughout the disturbances (*p* < .01; Figure 2). There was no relationship between how goby species changed their host use and whether they were host generalists or host specialists for either categorical metric (i.e., coral richness specificity, *p* < .5, and proportional coral specificity, *p* < .5). No patterns were observed regarding which specific coral species were occupied by host specialists or host generalists (e.g., host specialist *G. aoyagii* did not use the same coral species as host specialist *G. axillaris*) (Figure 2). However, gobies changed the extent to which they used a single coral species in the continuous host specialization metric (i.e., specificity continuum, *p* < .01). The sociality of gobies (whether they are social, a.k.a. live in groups, or asocial, a.k.a. live in pairs) was marginally affecting their host use (*p* = .049).

For the coral richness specificity metric, host specialists (i.e., *G. aoyagii*, *G. axillaris*, and *G*. cf. *bilineatus*) occupied 1–3 host species predisturbances, but each species occupied their own range of host species (Figure 2). Cyclones had minimal effects on host occupation, but there were marked changes after heatwaves. Postdisturbances, host specialists either disappeared or occupied more host species than previously observed (Figure 2). Of the three host specialists, *G*. aoyagii was the only species that was present after disturbances (2018), but it switched to being a host generalist occupying 5 coral species and then disappeared 3 years postdisturbances. On the other hand, *G. axillaris*, which had disappeared after disturbances (2018), was observed once again 3 years postdisturbance and became a generalist occupying five coral species. The other seven goby species were host generalists inhabiting between 5 and 10 coral host species predisturbances (Figure 2). Cyclones had minimal effect on host occupation, but heatwaves again caused noticeable changes. Postdisturbances, out of the seven host generalists, five goby species were still present and all, but *G. histrio* remained host generalists, although *G. histrio* was only observed 10 times (Figure 2). Even three years postdisturbances, generalists continued occupying a wide range of hosts, including *G. histrio* again, although another generalist *G. rivulatus* had disappeared (Figure 2). Therefore, there was no clear pattern in how host specialists and generalists responded using the coral richness specificity metric.

For the proportional coral specificity metric, species that were originally host specialists, since they used a single host at least 75% of the time predisturbances (i.e., *G. aoyagii*, *G. axillaris*, *G*. cf. *bilineatus*, *G. histrio*, *G. rivulatus*), occupied different coral species per goby species (Figure 2). Half of these remained specialists after cyclones, but then, only two species (*G. axillaris*, *G. histrio*) were observed 3 years postdisturbances and each varied in their level of specialty (Figure 2). Species that were originally host generalists, using a single host <75% of the time predisturbances (i.e., *G. brochus*, *G. erythrospilus*, *G. fuscoruber*, *G. oculolineatus*, *G. quinquestrigatus*), also occupied different coral species per goby species (Figure 2). Following cyclones, two species become host specialists (*G. brochus*, *G. oculolineatus*), whereas others remained generalists. Then, 3 years after disturbances, only three species (*G. brochus*, *G. erythrospilus*, and *G. quinquestrigatus*), and they used several coral species although *G. erythrospilus* became borderline host specialist (Figure 2). Therefore, there were no clear patterns observed in how host specialists and generalists responded with the proportional coral specificity metric.

**FIGURE 3:**
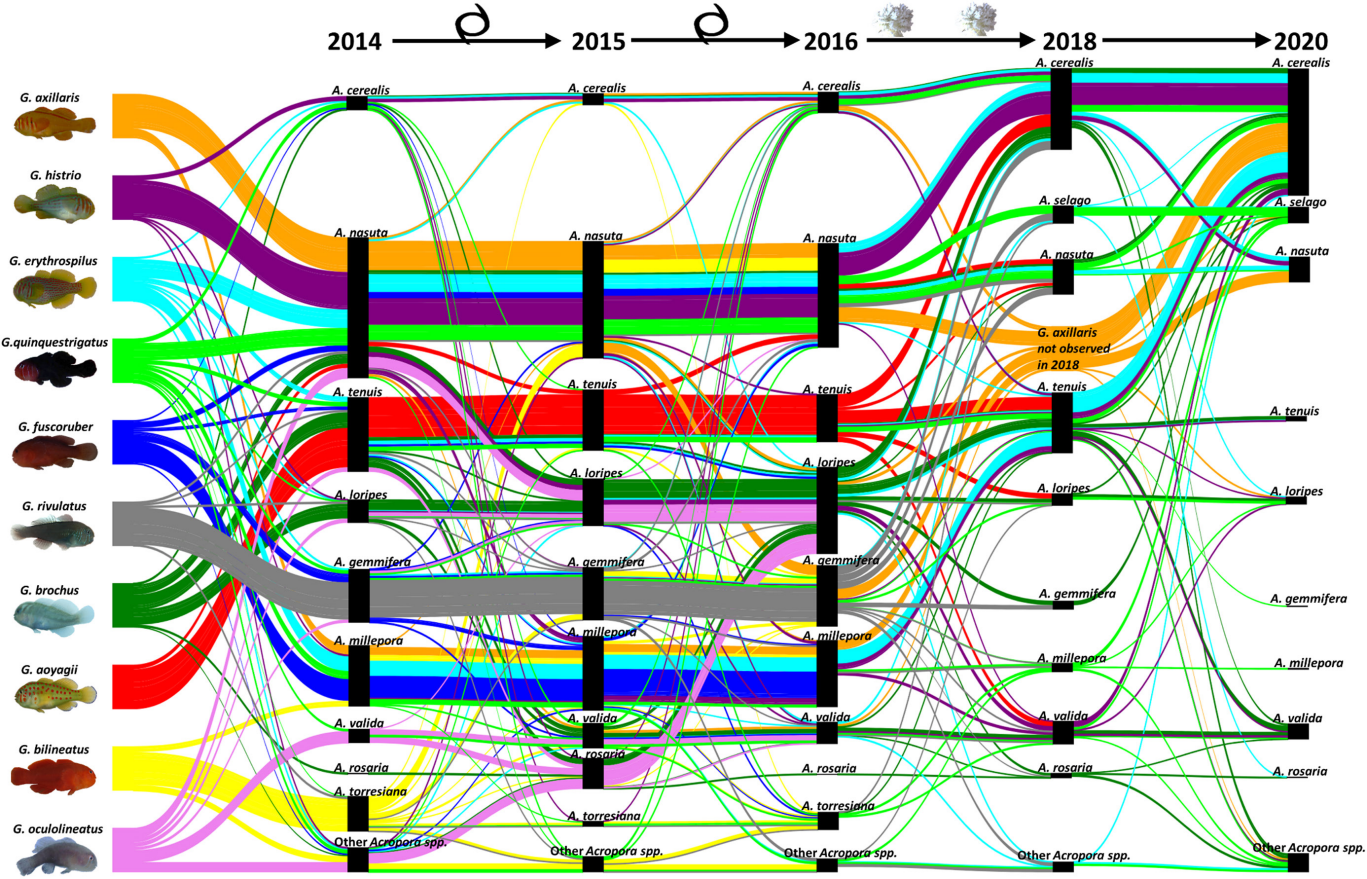
Goby occupancy of different corals at each time point. Data represent the frequency occupancy of *Gobiodon* goby species per *Acropora* coral species per time point. The top 10 most commonly occupied species of corals are illustrated here, and all other species are grouped in “other *Acropora* spp.” Each color represents a different goby species. The thickness of color bars is drawn to scale at each time point to represent the proportion of a specific coral species occupied by each goby species; that is, thicker bars represent corals more occupied by that goby species and thinner bars represent corals less occupied by that goby species. Corals are organized from top to bottom from those that increased in abundance postdisturbances (*A. cerealis* and *A. selago*) and to those that decreased in abundance postdisturbances (all other coral species).

The only metric that showed some patterns were in the specificity continuum, which calculated the proportion of occurrences that a goby species only occupied one coral species. Goby species that tended to occupy only one coral species used different coral species to goby species that tended to occupy several coral species. Regardless of being a host generalist or host specialist, each goby species occupied a single coral species in higher proportion over others (Figure 3). Gobies occupied a particular host between 25% and 90% of the time, although host specialists tended to occupy one host species more often than host generalists. Here presented are the single most occupied coral species per goby species, unless there was an even occupancy rate for two most occupied coral species for a goby species. For host specialists (using coral richness specificity), 90% of *G. aoyagii* occupied *A. tenuis*, 75% of *G. axillaris* occupied *A. nasuta*, and 75% *G*. cf. *bilineatus* occupied *A. torresiana* (Figures 2 and 3). For host generalists, 30% of *G. brochus* occupied *A. loripes* and 30% occupied *A. tenuis*, 40% of *G. erythrospilus* occupied *A. nasuta*, 50% of *G. fuscoruber* occupied *A. millepora*, 80% of *G. histrio* occupied *A. nasuta*, 25% of *G. oculolineatus* occupied *A. valida*, 35% of *G. quinquestrigatus* occupied *A. nasuta*, and 80% of *G. rivulatus* occupied *A. gemmifera* (Figures 2 and 3). Therefore, *A. nasuta* was the most commonly occupied host for four goby species, whether they were host specialists or generalists (Figure 3).

After the two cyclones, there was little change in the proportional occupancy of different coral hosts, suggesting that cyclones did not alter host specialization (Froehlich et al., 2021) (Figure 3). However after heatwaves, gobies shifted their host use, and often this shift mirrored the change in coral community. Many gobies switched from the previously popular *A. nasuta* to the newly abundant *A. cerealis* (Figures 1 and 3). Out of the remaining goby species postdisturbances, *Gobiodon aoyagii* began occupying *A. tenuis* and *A. cerealis* each 25% of the time, *G. histrio* switched to occupying the newly abundant *A. cerealis* 60% of the time, and three others (*G. brochus*, *G. erythrospilus*, and *G. rivulatus*) were also found more often in *A. cerealis* than previously observed (at least 20% of the time). The occupation of any particular host coral was not above 45% for any goby species after heatwaves, except for *G. histrio*.

Three years postdisturbances, there was little change in the number of hosts occupied by each goby species, but the majority of gobies were primarily occupying *A. cerealis* as it was the most abundant (Figures 1 and 3). *Gobiodon axillaris* was observed once again but switched host to *A. cerealis* 65% of the time (Figures 2 and 3). For *G. histrio* and *G. erythrospilus*, both species switched to *A. cerealis* (75% and 70%, respectively), others like *G. brochus* switched to *A. cerealis* albeit to a lesser extent (30%), and *G. quinquestrigatus* switched to using both *A. cerealis* (35%) and *A. selago* (30%). Accordingly, even 3 years postdisturbances, most gobies used *A. cerealis* over other coral species (Figure 3).

## 4 DISCUSSION

As multiple disturbances are becoming the norm, we find that mutualisms on coral reefs are not responding as a collective unit. Our 7‐year study shows that *Acropora* corals are faring far better than their goby inhabitants (genus *Gobiodon*) 3 years after back‐to‐back climatic events (two cyclones and two heatwaves) (Froehlich et al., 2021). However, not all coral species responded the same to disturbances, suggesting that habitat use plays a key role in the decline of gobies. Indeed, some goby species shifted their host use after disturbances, although that shift may be a potential downfall to their fitness in the long term, as they were not inhabiting their previously most occupied hosts (Caley & Munday, 2003; Munday, 2001). Accordingly, host use is a mechanism that likely explains why goby inhabitants are slower to recover than their coral hosts as shifting hosts in the short term may not always lead to goby population resilience in the long term.

Nine months postdisturbances, populations of corals and gobies were each devastated, but gobies declined at least three times more than corals, and most corals were devoid of gobies (Froehlich et al., 2021). After 3 years of recovery time, coral hosts became twice as abundant and speciose compared with predisturbances, although coral sizes were three times smaller than predisturbances. Reduced competition for space among corals may have allowed a surge in abundance within a few years of recovery, yet corals also had to compete with fast‐growing algae and high incidences of corallivory (Baird & Hughes, 2000; Penin et al., 2010). For gobies though, half of the species became rare or absent 3 years postdisturbances, including two previously abundant species, *G. fuscoruber* and *G. rivulatus*. There were four times fewer adult gobies compared with predisturbances. Gobies were never found in dead corals (Bonin et al., 2009). In addition, these gobies were living singly, which suggested even slower recovery since gobies need to live in pairs or groups to reproduce (Wong & Buston, 2013). A local recruitment failure may have occurred as a consequence of extreme heatwaves (Munday et al., 2008) and may explain why gobies lived singly as very few gobies were found after bleaching events. Alternatively, since corals remained very small, gobies may have been unable to pair and breed as they need larger corals to do so (Kuwamura et al., 1996). Gobies may be facing a population bottleneck (Sergio et al., 2018) due to the inability to form pairs over multiple years and breed to replenish recovering population. Alarmingly, 75% of corals no longer hosted gobies postdisturbances compared with just 5% predisturbances (Froehlich et al., 2021). No single coral species was ever occupied more than 60% of the time postdisturbances, whereas several species had previously occupied up to 100% of the time predisturbances. Even with 3 years of recovery time, 75% of corals were still devoid of gobies. Such a lag in goby population recovery is dire for the mutualism of corals and gobies. Coral‐dependent fishes are predicted to decline substantially with climate change (Buchanan et al., 2016), and gobies are a striking example of this phenomenon.

Given that habitat specialization likely plays a key role in the continued prevalence of coral and goby symbioses, our finding that half the gobies disappeared is a cause for concern. We categorized host specialization using three different metrics to assess different aspects of host use. Two of the metrics separated goby species into host specialists or host generalists using different methods, and yet neither metric could explain the changes in host use of gobies throughout climatic disturbances. With coral richness specificity metric in which we categorized goby species based on the number of corals they inhabited, we found that one‐third of the goby species inhabited just 2–3 host species predisturbances (i.e., host specialists), while others occupied a broader range of hosts (i.e., host generalists) (Dirnwöber & Herler, 2007; Munday, 2000; Munday et al., 1999). Two out of the three host specialists were absent 3 years postdisturbances, which suggests that host specialists may be less resilient to disturbances (Ainsworth & Drake, 2020; Dirnwöber & Herler, 2007; Hof et al., 2012). However, three out of seven host generalists also disappeared 3 years postdisturbances. With the proportional coral specificity metric, five goby species inhabited a single coral species at least 75% of the time predisturbances (i.e., host specialists), and five goby species inhabited a single coral species <75% of the time (i.e., host generalists). Within both categories, about half of the species disappeared 3 years postdisturbances, and some species switched from specialists to generalists and vice versa. Since the distinctions between host specialists and generalists, using either metric, did not provide any indication of how a goby species would respond to climatic disturbances, our findings suggest that categorical host specialization may not be an indicator of vulnerability to disturbances, as shown in terrestrial host symbioses like in plant–pollinator interactions in temperate forests (Vázquez & Simberloff, 2002). The extent and severity of disturbances and the differential susceptibility among specific corals may instead affect how particular species respond.

Albeit differences between host generalists and specialists not providing any indication of how a goby species would respond to climatic disturbances, we found that there were differences in the single most occupied coral species used by each goby species, that is, their host specificity continuum. The disappearance of half of the goby species mirrored the decline in their most occupied coral host species immediately after cyclones and heatwaves. Thus, despite being an advantage during stable periods, primarily occupying only one type of habitat may be a significant disadvantage during unstable periods (Feary, 2007; Munday, 2000). Even more alarmingly, many goby species stayed rare or disappeared despite the host species they occupied predisturbances increasing in abundance 3 years postdisturbances. For example, *G. fuscoruber* and *G. rivulatus* disappeared even though their previously most occupied hosts, *A. millepora* and *A. gemmifera*, respectively, reappeared in higher abundance 3 years postdisturbances. Yet, *G. axillaris*, which primarily occupied *A. nasuta*, initially disappeared 1 year postdisturbances but then returned 3 years postdisturbances and switched to occupying *A. cerealis* as it became more abundant. Our findings suggest that some gobies exhibit host plasticity with regard to the single most occupied host species and that there is no clear advantage of being a host specialist or host generalist.

Whether gobies are able to remain on their previously occupied host species or shift to newly available host species is key to their recovery. Since we observed shifts in host use for several goby species, postsettlement processes that are likely influencing these shifts. Gobies that survived disturbances may shift their hosts as adults, although such movement has high predation risks and rarely occur after individuals have settled into a coral host (Bonin et al., 2009; Froehlich et al., 2022; Munday et al., 1998; Nakashima et al., 1996; Wong & Buston, 2013). More likely, host shifts seen at a population level occurred from settling individuals selecting different coral hosts postdisturbances based on the coral distribution. We found that goby populations overall decreased substantially with very few adult gobies being observed postdisturbances. Instead, more juveniles were observed 3 years postdisturbances than predisturbances, suggesting new recruits drove the shifts in host use.

Other mechanisms of the biology and ecology of coral‐dwelling gobies may be adding to their limited recovery from climatic disturbances. Due to a larval dispersal stage, coral reef fishes have the potential for larvae to be supplied from locations far away through stochastic replenishment (Green et al., 2015; Hing et al., 2018; Munday et al., 1998). However, broadscale disruptions to larval supply are likely occurring as climatic disturbances have broadscale reach, and such disruptions have already been shown in *Acropora* corals as well (Hughes et al., 2019). Coral‐dwelling gobies may even experience higher disruptions to larval supply compared with corals as their larvae may be settling closer to their natal habitat than expected, as seen in other coral reef fishes (Gerlach et al., 2007; Rueger et al., 2020, 2021; Selwyn et al., 2016). Limited recovery in coral‐dwelling gobies may also be a consequence of their social tendencies to live in pairs or groups depending on the species (Hing et al., 2018). Out of the 10 *Gobiodon* species that we focused on in our study, two were known to live in groups (*G. fuscoruber* and *G. rivulatus*), and both species became extremely rare after disturbances. Our study found a marginally significant signal that the sociality of gobies may impact their host use. However, habitat constraints may explain a decline in group‐living species, as larger corals can house more goby individuals (Hing et al., 2018). Since corals were substantially smaller after disturbances, group‐living species were likely at a disadvantage. Accordingly, it is possible that group‐living species are less resilient to climatic disturbances, although this needs to be studied further with more species and at more locations.

Although unoccupied corals are on the rise and may be able to survive in the short term, a prolonged lack of mutualistic goby partners may increase their vulnerability to external threats in the long term since gobies provide beneficial services to corals (Chase et al., 2018; Chase, Pratchett, McWilliam, et al., 2020; Dirnwoeber & Herler, 2013; Dixson & Hay, 2012; Penin et al., 2010). However, it is possible that other goby species may shift hosts in the short‐term, given the host plasticity observed in some goby species in this study. Such host shifts may increase coral resilience but potentially decrease goby fitness, since goby growth rates are higher in certain coral species (Caley & Munday, 2003; Munday, 2001). It is important to note that several goby species switched from *A. nasuta* to the newly abundant *A. cerealis*, which are very similar in morphology and may potentially allow gobies to grow at similar rates if the morphology of the coral is the only limiting factor to their growth. More coral species were observed 3 years postdisturbances than at any other time point, and yet surviving goby species all shifted to primarily using the same coral species, *A. cerealis*. However, some gobies species that later switched to *A. cerealis* had initially occupied morphologically different corals like *A. loripes* and *A. tenuis* as they have higher survival rates in these corals (Munday, 2001). Certain inhabiting species may also be less effective at promoting the resilience of hosts (Douglas, 1998; Visser & Gienapp, 2019), which has yet to be studied in host‐animal symbioses in the marine environment but has been observed in plant–animal interactions like acacia ant–plant mutualisms following fire disturbance (Sensenig et al., 2017) and bark beetle‐fungus symbioses with thermal stress (Six et al., 2011). While the capacity for host shifts may promote the initial short‐term survival of both partners, the long‐term fitness of both gobies and corals may decline over time unless other coral symbionts fill the symbiont niche (Bonin, 2012; McKeon et al., 2012; Thompson et al., 2015). Inhabiting fishes are particularly important for the resilience of their coral host to thermal stress (Chase et al., 2018), as is also seen in *Populus tremuloides* host plants with ants and aphids (Marquis et al., 2014). As coral‐dwelling fish reduce bleaching susceptibility and impacts of sedimentation of coral hosts (Chase et al., 2018; Chase, Pratchett, McWilliam, et al., 2020), their decline may potentially spell disaster for coral resilience Future studies should quantify recovery rates of corals with or without fish inhabitants to further determine how much coral‐dwelling fishes contribute to the resilience of corals.

Our study is an early warning sign that mutually symbiotic partners may not recover at similar rates, and, while the capacity for host plasticity may be key for immediate survival, it may not improve resilience to future environmental and other stressors. Given that disturbances are occurring more frequently than ever before (Hughes et al., 2018; Turner, 2010), the mutualism between coral hosts and gobies may not be able to persist after continued disturbances, leaving both organisms susceptible to additional stress. Mutualism breakdowns are being observed in various environments, for example, seagrass beds (De Fouw et al., 2016), tidal environments (Dunkley et al., 2020), and myrmecophyte habitats (Kiers et al., 2010). As mutualisms are predicted to change drastically moving forward (Kiers et al., 2010), such changes could even have knock‐on effects on ecosystem stability (Kiers et al., 2010; Six et al., 2011; Turner, 2010; Wilson et al., 2006). Whether symbionts exhibit host plasticity to changing environments is a key factor in understanding the potential resilience of corals and coral reef ecosystems to climate change.

## AUTHOR CONTRIBUTIONS

**Catheline Y. M. Froehlich:** Conceptualization (lead); data curation (lead); formal analysis (lead); funding acquisition (equal); investigation (lead); methodology (equal); project administration (lead); validation (lead); visualization (lead); writing – original draft (lead); writing – review and editing (lead). **O. Selma Klanten:** Conceptualization (supporting); investigation (supporting); methodology (supporting); supervision (equal); writing – review and editing (equal). **Martin L. Hing:** Investigation (equal); methodology (equal); writing – review and editing (equal). **Mark Dowton:** Funding acquisition (equal); supervision (supporting); writing – review and editing (equal). **Marian Yi‐Ling Wong:** Conceptualization (supporting); funding acquisition (equal); investigation (supporting); methodology (equal); resources (lead); supervision (lead); writing – review and editing (equal).

### OPEN RESEARCH BADGES

This article has earned Open Data and Open Materials badges. Data and materials are available at doi: 10.5063/F1639N69.

## Supporting information


Appendix S1

## Data Availability

The dataset and methods are published at doi: 10.5063/F1FJ2F8Q.
