# Supplementary material for: Delayed recovery and host specialization may spell disaster for coral‐fish mutualism
Source: Ecol Evol. 2023 Jun 22;13(6):e10209. doi: 10.1002/ece3.10209 (PMC10285627; doi:10.1002/ece3.10209)
Supplement: Supplementary file 1 — Appendix S1 [file ECE3-13-e10209-s001.pdf]

Appendix for Manuscript:

Delayed recovery and host specialization may spell disaster for coral-fish mutualism

By Catheline Y.M. Froehlich, O. Selma Klanten, Martin L. Hing, Mark Dowton, and Marian Y.L. Wong

Additional figures supporting the manuscript.

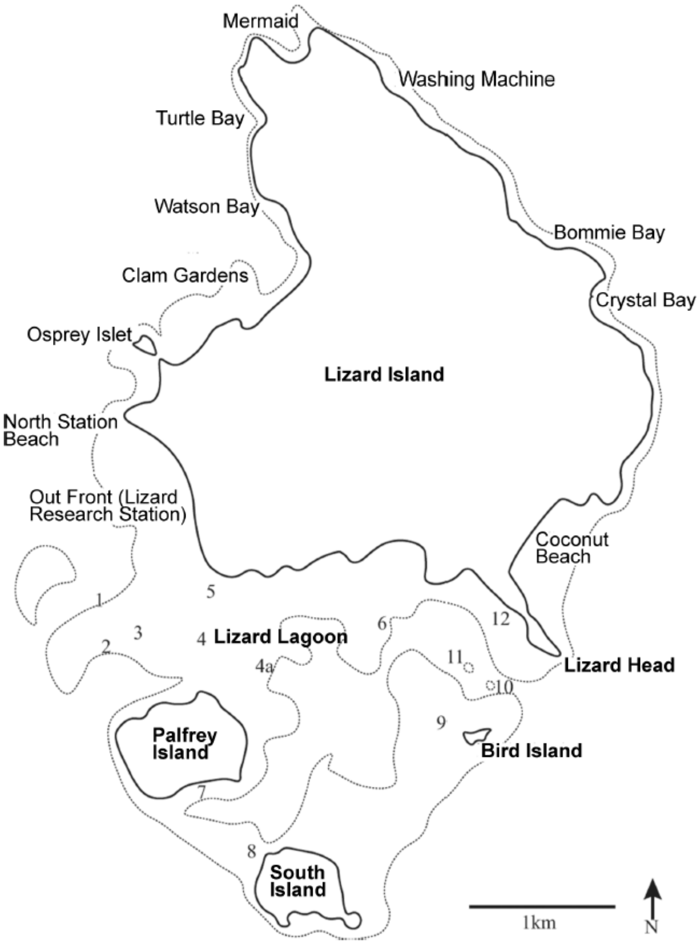

Appendix F1. Map of Lizard Island, QLD (-14.687264, 145.447039). Numbers represent discrete sites as follows: Big Vickey's Reef (1); Vickey's Reef (2); Horseshoe Reef (3); Palfrey Reef (4±4a); Loomis Reef (5); Trawler (6); Picnic Beach (7); Ghost Beach (8); Bird Island Reef (9); Entrance Bommie (10); Bird Bommie (11); Coconut Head Reef (12).

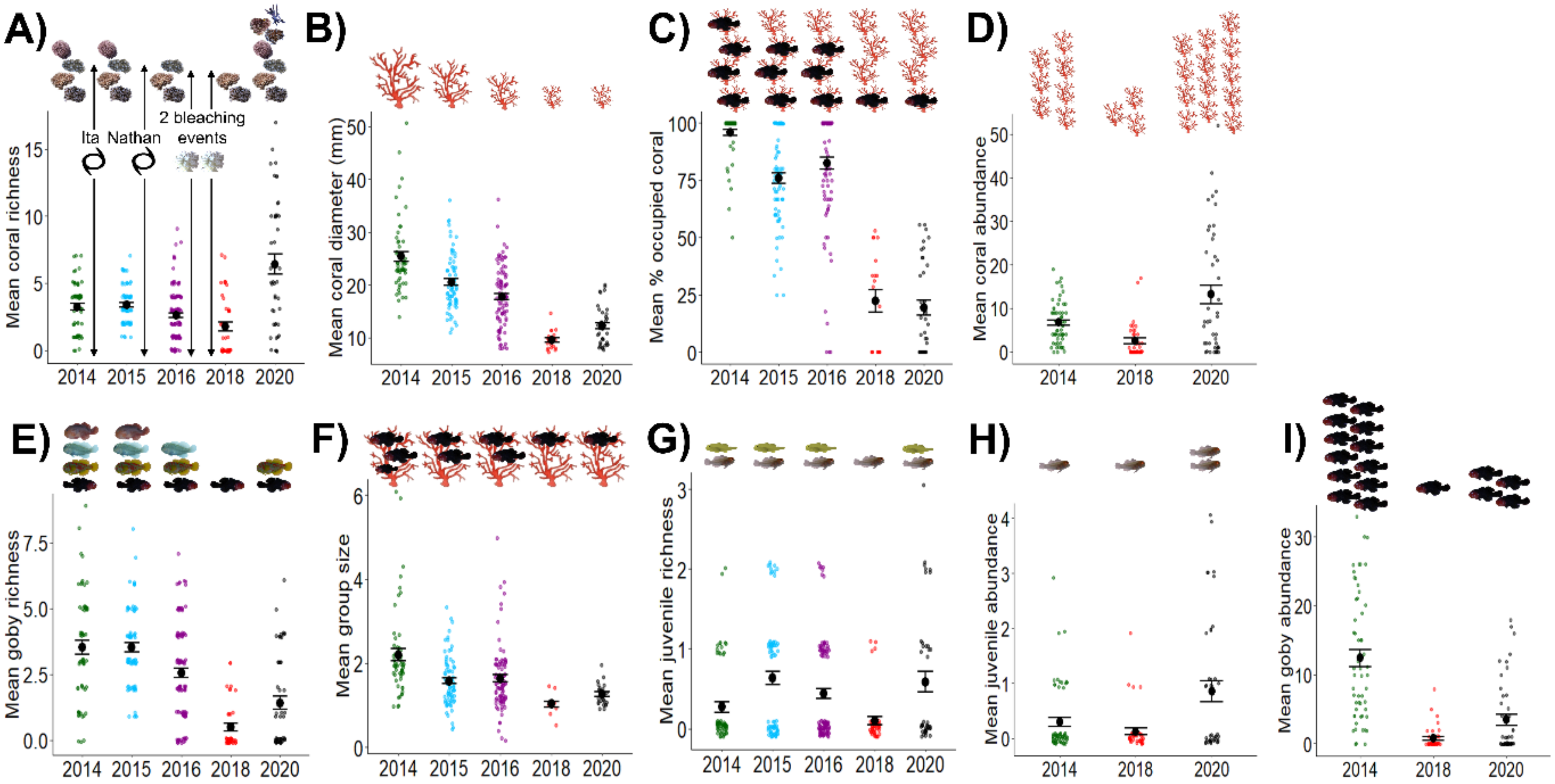

Appendix F2. Multiple disturbances affect the populations of *Acropora* corals and *Gobiodon* gobies. (A) Coral richness, (B) diameter,(C) occupancy by gobies, and (D) abundances, (E) adult goby richness and (F) group size, (G) juvenile goby richness, and the abundance of (H) juvenile gobies, and (I) adult gobies per transect before and after each cyclone (black spiral symbol), after heatwaves/bleaching (white corals), and 3 yr post-disturbances. Abundances (D,H,I) were only calculated in 2014, 2018, and 2020 due to differences in transect methodology. Symbols above data points depict changes in means as identified through post-hoc testing. Error bars are standard error.

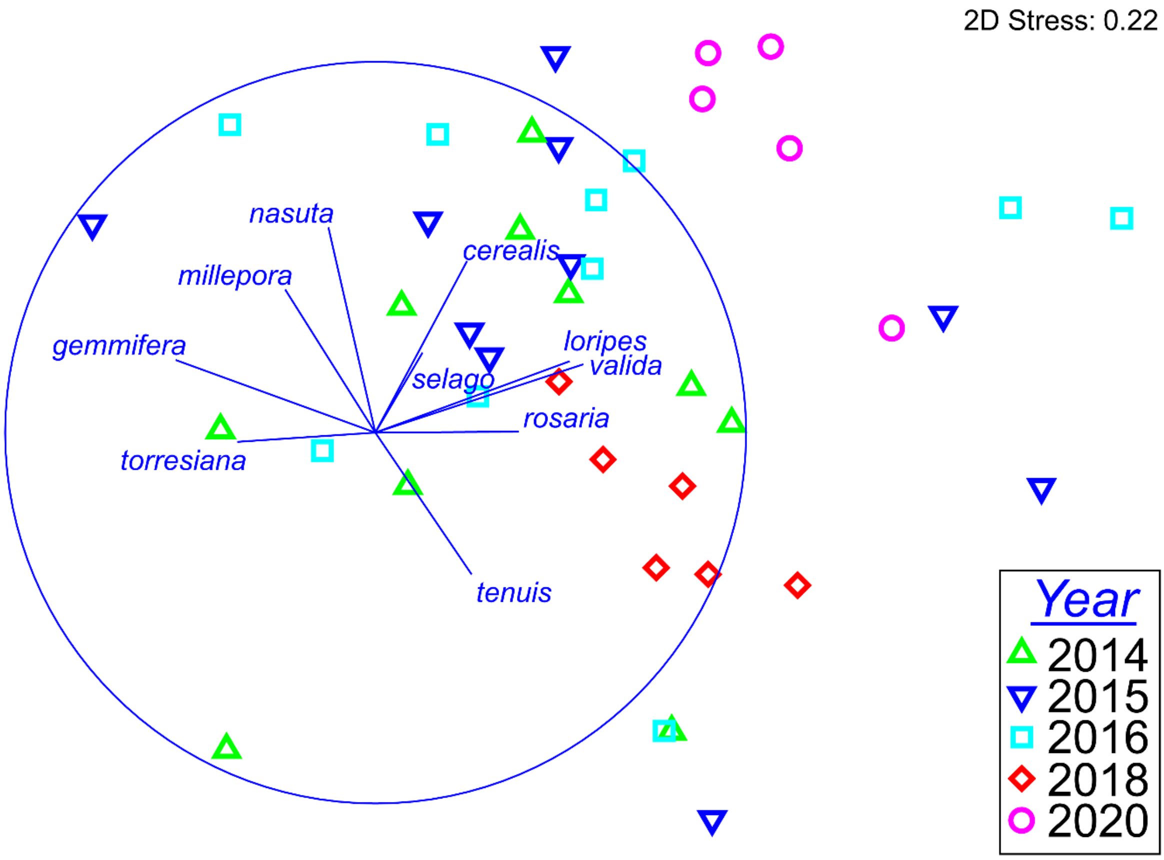

Appendix F3. Coral assemblages inhabited by each goby species following multiple disturbances. Multidimensional scaling plot illustrating surveys were completed: before (2014), after Cyclone Ita (2015), after cyclone Nathan (2016), after two back-to-back bleaching events (2018), and 3 yr after disturbance (2020). Each point represents the diversity of corals used by each goby species within a single survey time period. Points closer together are more similar in coral assemblages than points further apart. Overlayed are the direction in which *Acropora* coral species are most abundant, and the closer the trajectory is to reaching the outer circle, the most abundance of that species is explained in that direction.

Appendix T1. Output of all statistical analyses. GLMM = generalized linear mixed model, LMM = linear mixed model, PERMANOVA = permutational analysis of variance

Response Variable	Model	Predictor variable	Factor Type	df	Model Statistic	Test-value	p-value	R-squared
Coral Richness	GLMM	Year	fixed	3	χ^2 (chi-	87.395	< 0.0001*	0.185
	poisson	Site	random		squared)			marginal
	zero-inflated							
					Sample size	n = 326		
					Outliers removed	n = 9		
Average Coral Diameter	LMM	Year	fixed	3	F-value	64.158	< 0.0001*	0.562
log-transformed		Site	random					conditional
					Sample size	n = 283		
					Outliers removed	n = 4		
Percent Occupied	LMM	Year	fixed	4	F-value	132.21	< 0.0001*	0.723
Corals		Site	random					conditional
					Sample size	n = 283		
					Outliers removed	n = 6		
Adult Goby Richness	GLMM	Year	fixed	7	χ^2 (chi-	115.35	< 0.0001*	0.5
	poisson	Site	random		squared)			marginal
	zero-inflated							
					Sample size	n = 326		
					Outliers removed	n = 4		
Average Goby	LMM	Year	fixed	4	F-value	10.471	< 0.0001*	0.194
Group Size		Site	random					conditional
log-transformed								
					Sample size	n = 283		
					Outliers removed	n = 3		
Juvenile Goby Richness	GLMM	Year	fixed	7	χ^2 (chi-	25.843	< 0.0001*	0.21
	poisson	Site	random		squared)			marginal
	zero-inflated							
					Sample size	n = 326		
					Outliers removed	n = 11		
Coral Abundance	GLMM	Year	fixed	3	χ^2 (chi-	259.68	< 0.0001*	0.306
	poisson	Site	random		squared)			marginal
	zero-inflated							
					Sample size	n = 146		
					Outliers removed	n = 5		
Juvenile Goby Abundance	GLMM	Year	fixed	5	χ^2 (chi-	18.898	< 0.0001*	0.371
	poisson	Site	random		squared)			marginal
	zero-inflated							
					Sample size	n = 146		
					Outliers removed	n = 5		
Adult Goby Abundance	GLMM	Year	fixed	3	χ^2 (chi-	198.36	< 0.0001*	0.674
	poisson	Site	random		squared)			marginal
	zero-inflated							
					Sample size	n = 146		
					Outliers removed	n = 5		
Common 10 coral community	PERMANOVA	Year	fixed	2	pseudo-F	7.958	0.0001*	N/A
		Site	random	16		3.401	0.0001*	
		Year*Site		32		1.874	0.0001*	
		Residual		95				
					Sample size	n = 146		
Common 10 goby community	PERMANOVA	Year	fixed	2	pseudo-F	10.963	0.0001*	N/A
		Site	random	17		3.465	0.0001*	
		Year*Site		31		1.719	0.0001*	
		Residual		95				
					Sample size	n = 146		
Corals Used by Gobies	PERMANOVA	GobySpp	fixed	9	pseudo-F	5.815	0.0001*	N/A
		Year	fixed	4		9.58	0.0001*	
		GobySpp*Year	fixed	27		1.288	0.005*	
		Residual		124				
					Sample size	n = 165		
Corals Used by Gobies	PERMANOVA	Specificity Continuum	covariable	1	pseudo-F	5.964	0.0001*	N/A
		Year	fixed	4		9.538	0.0001*	
		GobySpp	fixed	8		5.817	0.0001*	
		GobySpp*Year		27		1.288	0.005*	
		Residual		124				
					Sample size	n = 165		
Corals Used by Gobies	PERMANOVA	Coral Richness Specificity	fixed	1	pseudo-F	0.915	0.5124	N/A
		Year	fixed	4		6.189	0.0001*	
		GobySpp(Coral Rich Spec)	fixed	8		5.934	0.0001*	
		Coral Rich Spec*Year	fixed	4		1.466	0.0646	
		GobySpp*Year	fixed	23		1.21	0.038*	
		Residual		124				
					Sample size	n = 165		
Corals Used by Gobies	PERMANOVA	Proportional Coral Specificity	fixed	1	pseudo-F	0.81	0.587	N/A
		Year	fixed	4		5.829	0.0001*	
		GobySpp(Prop Coral Spec)	fixed	8		5.898	0.0001*	
		PropCoral Spec*Year	fixed	4		1.176	0.2474	
		GobySpp*Year	fixed	23		1.252	0.0197*	
		Residual		124				
					Sample size	n = 165		

Appendix T1. Output of all statistical analyses. GLMM = generalized linear mixed model, LMM = linear mixed model, PERMANOVA = permutational analysis of variance

Response Variable	Model	Predictor variable	Factor Type	df	Model Statistic	Test-value	p-value	R-squared
Corals Used by Gobies	PERMANOVA	Sociality Index	fixed	1	pseudo-F	2.295	0.0491*	N/A
		Year	fixed	4		6.515	0.0001*	
		GobySpp(Sociality)	fixed	8		5.217	0.0001*	
		Sociality*Year	fixed	3		2.104	0.004*	
		GobySpp*Year	fixed	24		1.138	0.1139	
		Residual		124				
					Sample size	n = 165		
